# Commentary: The Extent and Consequences of P-Hacking in Science

**DOI:** 10.3389/fpsyg.2020.581910

**Published:** 2020-09-30

**Authors:** Juan Botella, Manuel Suero

**Affiliations:** Faculty of Psychology, Universidad Autónoma de Madrid, Madrid, Spain

**Keywords:** questionable practice, p-hacking, *p*-values distribution, cross disciplinary, scientific hypotheses

Head et al. (2015) employed text mining procedures to extract significant *p*-values (operationally defined as *p* < 0.05) reported in many thousands of articles from a variety of scientific disciplines. Their goal was to assess the extent and consequences of *p*-hacking, a set of questionable research practices aimed at forcing research results to be statistically significant (John et al., 2012; Earp and Trafimow, 2015). They concluded that there is clear evidence of *p*-hacking, although its quantitative impact on the meta-analytical estimation of effect size (*ES*) is small.

What caught our attention was what seemed to be a strikingly high degree of similarity among the empirical distributions of *p*-values across disciplines with very distant study domains. When a true null hypothesis is tested, the distribution of *p*-values is expected to be uniform. The degree to which the true parametric value deviates from the hypothesized null value (the *ES*), is reflected in the degree to which the distribution of *p*-values deviates from a uniform distribution, thus showing asymmetry. Specifically, if the effect-size is non-zero, the expected distribution of *p*-values from which replications are sampled should be exponential, with an upper limit at 0.0, and a positive skew extending downward to 0.05 and beyond. We have derived a figure from Table 1 of Head et al. (2015), rearranging the frequencies into three intervals. The figure shows the percentages of *p*-values falling within each interval for the 14 disciplines studied. There is a high degree of similarity among these observed *p*-values across disciplines.

One might naively expect that in such disparate scientific disciplines the empirical distributions of *p*-values would be more varied. We discuss some alternative explanations to account for this (to us) surprising result.

The first explanation relates to the *similarity of methodologies* across disciplines. A large majority of these disciplines employed hypothesis testing through a hybrid of the Neyman-Pearson and Fisher methods. We could well be facing a scenario that results from the general application of a common statistical methodology. From this explanation, it is the nature of the methodology itself which, when applied correctly, leads to the observed convergence. The different scientific disciplines employ very similar statistical models, typically based on the general linear model, so it might well be expected that similar results. This explanation implies that the average *ES* of the phenomena under study are also very similar, or that they work with sample sizes that, in combination with the mean *ES* of each field, give rise in the long run to statistical tests with close average levels of power. According to this explanation there is nothing special about this convergence; it is the very nature of the method of analysis that inevitably leads to such cross-disciplinary uniformity.

The second explanation concerns the *similarity of scientists' questionable research practices*. The observed convergence could be the by-product of such practices, together with the collaboration of the reviewers of the journals, who often only look at whether the result is significant, without paying attention to other issues as the power or the *ES*. A mixture of proper and questionable research practices that are similar across disciplines could also lead to the results reported by Head et al. (2015) as shown in Figure 1. It has nothing to do with the phenomena that are studied in each discipline; rather, the system of reinforcements and rewards of science leads to the same distributions of *p*. The starting point is the rigorous and neutral scientific practice that is learned during research training. The reinforcement system then shapes the behavior of scientists, reinforcing some tendency toward certain deviant or questionable practices (Giner-Sorolla, 2012). Because contingencies are similar across disciplines, the shaped behavior ends up being very similar. From this perspective, the empirical distribution of *p*-values is nothing more than a reflection of the combination of rigorous and questionable behaviors that in each discipline maximize the rewards in our professional scientific systems.

**Figure 1:**
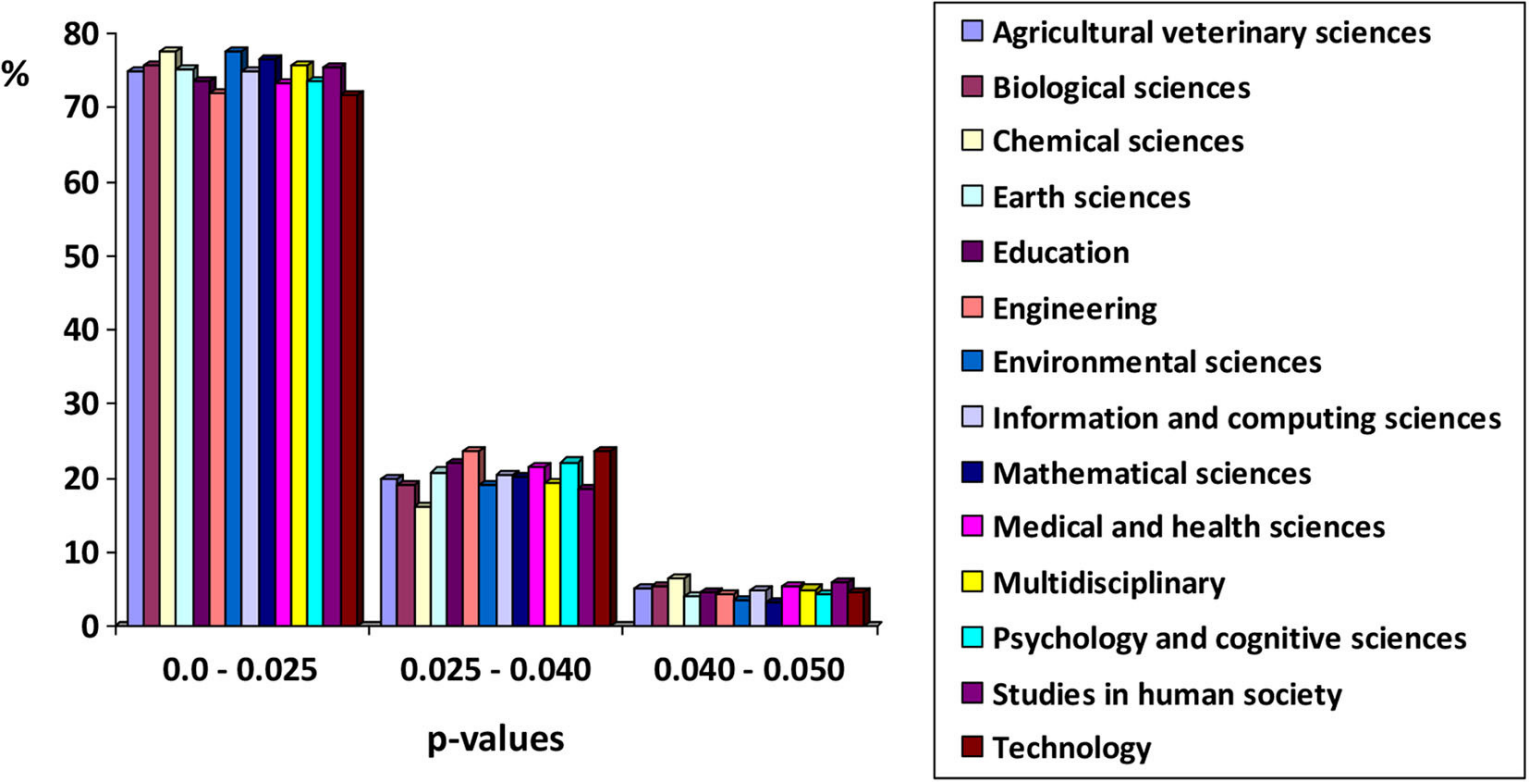
Empirical distributions of significant *p*-values (*p* < 0.05) across 14 disciplines. Prepared from the data in Table 1 of Head et al. (2015).

The third explanation has to do with the *similarity of scientific hypotheses*. It is also a psychological explanation but based on the cognitive processes of the scientists themselves. Essentially, the problems faced by researchers in the different fields imply an active search for similar *ES* values. Again, the reinforcement and rewards systems come into play. However, what is being shaped is the choice of scientific problems to investigate. The need to publish and obtain research funding drives scientists to select those questions that optimize professional performance. The phenomena that attract the attention of scientists are those that are most likely to demonstrate large differences from a null effect. Those phenomena that are likely to produce smaller *ESs* are less worthy of study as they are also less likely to lead to ultimate success and recognition. The selection of Research Topics might be the result of cognitive processes that are shaped according to the law of effect. The idea is that certain questions (with their answers and their *ESs*) are constrained to some extent by the social recognition system of science. These are questions that involve Research Topics that differ in important ways from what is already known. Significant results are more highly reinforced and are more likely to be repeated. The difference from the second explanation is that what is shaped here is not tendency toward questionable research practices, but selective attention to a class of genuine problems; the choice of questions that in the end are professionally more efficient. Of course, all this would not necessarily be reflected in conscious and declarative cognitive processes.

Perhaps the correct explanation is one of the three discussed here or possibly different ones, or any combination of them. The similarity of the *p*-value distributions invites us to think about what the different disciplines have in common. One of the common elements is the scientists themselves and their behaviors. Whether questionable or not, the behavior of scientists, in interaction with our scientific environment, has become an exciting and important object of study. The great convergence in the distributions of *p*-values is a product of human behavior that deserves to be studied and explained.

## Author Contributions

JB and MS have made substantial, direct and intellectual contribution to the work, and approved it for publication. Both authors contributed to the article and approved the submitted version.

## Conflict of Interest

The authors declare that the research was conducted in the absence of any commercial or financial relationships that could be construed as a potential conflict of interest.
